# Supplementary material for: Association of polymorphic markers of genes FTO, KCNJ11, CDKAL1, SLC30A8, and CDKN2B with type 2 diabetes mellitus in the Russian population
Source: PeerJ. 2017 Jul 13;5:e3414. doi: 10.7717/peerj.3414 (PMC5511504; doi:10.7717/peerj.3414)
Supplement: Supplemental Information 2 [file peerj-05-3414-s002.docx]

Table S1. Sequence of primers, fluorescent probes, and specific features of the amplification of the polymorphic regions of genes *FTO*, *KCNJ11*, *SLC30A8,* *CDKN2B,* and *CDKAL1*

| Gene | Polymorphic  marker | Genotyping method | Sequence  of primers, 5`−3` | Sequence  of probes, 5`−3` | Annealing temperature, ^°^С |
| --- | --- | --- | --- | --- | --- |
| *FTO* | *rs8050136* | TaqMan | gcttcatagcctagtcta  gcttcatagcctagtcta | cactgtggcaataaatatctgagc  cactgtggcaatcaatatctgagc | 58 |
|  | *rs7202116* | TaqMan | gcctaatgttgaaatctca  gaacctccatcattcacta | taactaatcatataaacatctttcatcttagactg  taactaatcatataaacgtctttcatcttagactg | 58 |
|  | *rs9930506* | TaqMan | gtgtgatccaatattaggg  ctaggtatgtatcaacttca | aagggacatactacatgaattactaatatc  aagggacatactacgtgaattactaatatc | 60 |
| *KCNJ11* | *rs5219* | TaqMan | gaggaatacgtgctgaca  tgcctttcttggacacaa | aggaccctgccaagcccaggta  aggaccctgccgagcccaggta | 62 |
| *SLC30A8* | *rs13266634* | TaqMan | tctccctgtgcttctttatc  gtgagtgagtgcatcgta | agcagccagccgggacagcc  agcagccagctgggacagcc | 60 |
| *CDKN2B* | *rs10811661* | TaqMan | aagcgttcttgccctgtc  ggtaggaggagccagaaga | cctccagctttagttttcccatgacagtaagtct  cctccagctttagttttctcatgacagtaagtct | 60 |
| *CDKAL1* | *rs7756992* | TaqMan | tttgacaattaatattccc  ttttaacacacaagaatc | tgtattttagttttagatctacagtt  tgtattttagttttggatctacagtt | 54 |
|  | *rs9465871* | TaqMan | gagtgatcagctgtgtaa  ccagttccctattgacaa | tgttgctgagaaactgagttagatgaa  tgttgctgagaaattgagttagatgaa | 55 |
|  | *rs7754840* | TaqMan | ccagatataccaccaaaa  acctcagtcaataacaga | aatgttggaaacgttgacttgat  aatgttggaaaggttgacttgat | 55 |
|  | *rs10946398* | TaqMan | tataattaggttgaactggtt  gtaagacaagtgttctgatat | tttagtatcgttatgctgtcattgc  tttagtatcgttctgctgtcattgc | 53 |
